# Decreased Tuberculosis Incidence and Declining Clustered Case Rates, Madrid

**DOI:** 10.3201/eid1410.080233

**Published:** 2008-10

**Authors:** Jesús Iñigo, Araceli Arce, Elia Palenque, Darío García de Viedma, Fernando Chaves

**Affiliations:** Consejería de Sanidad Comunidad de Madrid, Madrid, Spain (J. Iñigo, A. Arce); Hospital 12 de Octubre, Madrid (E. Palenque, F. Chaves); Hospital Gregorio Marañón, Madrid (D. Garcia de Viedma); Centro de Investigación Biomédica en Red Enfermedades Respiratorias, Madrid (D. Garcia de Viedma)

**Keywords:** Tuberculosis transmission, molecular epidemiology, tuberculosis prevention and control, dispatch

## Abstract

To determine effect of recent tuberculosis transmission rates on incidence rates, we conducted 2 prospective population-based molecular epidemiologic studies in Madrid during 1997–1999 (4% immigrants) and 2002–2004 (14.9% immigrants). Case rates decreased in association with declining clustered case rates among Spanish-born persons. New strains were introduced through immigration.

During the past decade in Madrid, Spain, tuberculosis (TB) case rates have decreased substantially while the proportion of foreign-born persons with TB has increased (2.6% in 1994 to 33.7% in 2003) (*1*). We used a combination of genotyping and conventional epidemiologic investigation to determine the extent to which the decline in incidence of TB in Madrid was affected by changes in rate of recent transmission of disease.

## The Study

We conducted 2 prospective population-based studies of TB patients in 3 urban districts of Madrid over 2 separate periods: 1997–1999 (population 455,050; 4% immigrants) and 2002–2004 (population 488,518; 14.9% immigrants). We included all TB patients in these 3 districts and used the same methods for both studies. All clinical samples were sent to the Microbiology Department of Hospital 12 de Octubre for TB testing. All patient information was collected by using a standardized protocol based on the Regional Registry of Tuberculosis Cases in Madrid.

Clinical specimens were processed according to standard methods. DNA fingerprinting was performed by restriction fragment length polymorphism (RFLP) analysis with the insertion sequence IS*6110*. Computer-assisted analysis was performed by using Bionumerics software (Applied Maths, Kortrijk, Belgium). All strains with <6 copies of IS*6110* were spoligotyped. Within each study period, cases were considered to be clustered if common RFLP patterns containing >6 indistinguishable IS*6110* bands or patterns containing <5 indistinguishable IS*6110* bands and identical spoligotyping patterns were found.

For each period we calculated average annual TB case rates and rates of clustered and nonclustered cases. Census populations of each district were used as denominators. Statistical analyses used Epi Info version 3.3 (Centers for Disease Control and Prevention, Atlanta, GA, USA) and Epidat (Pan American Health Organization, Washington, DC, USA).

Case numbers were 412 during 1997–1999 (average incidence 30.2 cases/100,000) and 377 during 2002–2004 (average incidence 25.7/100,000); p<0.001 (Table 1). The 150 (19%) foreign-born patients were from 20 different countries. Date of arrival in Spain was available for 81 (54%); 78 (96.3%) had been in Spain <5 years before TB diagnosis, and 39 (48.2%) diagnoses were made within the first 2 years of residence. Median time from arrival to onset of treatment was 30.1 months (25th–75th percentiles 8.0–50.7).

**Table 1:** Characteristics of tuberculosis patients during 2 periods in 3 urban districts of Madrid, Spain

Characteristic	Years		p value
	1997–1999, no. (%), n = 412	2002–2004, no. (%), n = 377	
Gender			
Male	281(68.2)	244 (64.7)	
Female	131 (31.8)	133 (35.3)	0.33
Age group, y*			
<35	178 (43.2)	159 (42.2)	
35–64	151 (36.7)	133 (35.3)	
>65	78 (18.9)	84 (22.3)	0.55
Foreign-born			
Yes	22 (5.3)	128 (34.0)	
No	390 (94.7)	249 (66.0)	<0.001
HIV status			
Positive	106 (25.7)	46 (12.2)	<0.001
Negative or unknown	306 (74.3)	331 (87.8)	
Injection drug use			
Yes	81(19.7)	33 (8.8)	<0.001
No or unknown	331 (80.3)	344 (91.2)	
Localization of tuberculosis			
Pulmonary	292 (70.9)	275 (72.9)	0.57
Extrapulmonary or unknown	120 (29.1)	102 (27.1)	

*Age unknown for 5 patients in 1997–1999 and 1 patient in 2002–2004.

In the first period, 328 cases (79.6%) were confirmed by isolation of *Mycobacterium tuberculosis.* RFLP analysis was performed on 212 isolates (64.6% of culture-positive cases and 51.4% of TB cases), and 95 of the 212 isolates (44.8%) shared an RFLP pattern with at least 1 other case and could be grouped into clusters. Risk factors associated with clustering were age <35 years (odds ratio [OR] 3.3, 95% confidence interval [CI] 95% 1.8–6.1; p<0.001), injection drug use (OR 2.1, CI 95% 1.0–4.6; p = 0.04), and previous imprisonment (OR 3.1, CI 95% 1.4–7.3; p = 0.004) (*2*). In the second period, 291 cases (77.2%) were confirmed by isolation of *M. tuberculosis.* RFLP analysis was performed on 201 isolates (69.1% of culture positive-cases and 53.3% of TB cases), and 64 of the 201 patients (31.8%) were clustered, the only statistically significant risk factor for which was birth in Spain (OR 2.2, CI 95% 1.1–4.6; p = 0.027). Contact investigation was performed for 56% and 70% during each period, respectively (p = 0.01). No statistically significant differences were found between the groups of patients whose isolates were and were not available for molecular typing in each period, except during the first period a greater proportion of pulmonary rather than extrapulmonary isolates was available for fingerprinting (84.4% vs. 71.6%, p<0.05).

Comparison between the 2 study periods showed that the decline in overall case rate was associated with a decrease in the rate of clustered cases from 7.0/100,000 to 4.4/100,000 (p<0.001) (Table 2). The nonclustered case rates did not statistically differ between the 2 periods (p = 0.45). Overall incidence of TB among the Spanish-born population decreased from 29.9/100,000 to 20.0/100,000 (p<0.001), among clustered (p<0.001) and nonclustered cases (p = 0.04). Despite no significant change in the overall case rate among the foreign-born population (40.0 vs. 58.7/100,000, p = 0.12), the rate of nonclustered cases increased significantly between the 2 periods (p = 0.02).

**Table 2:** Overall and clustered tuberculosis case rates during 2 periods in 3 urban districts of Madrid, Spain*

Characteristic	Case rate/100,000 persons†							
	Overall	p value		Clustered	p value		Nonclustered	p value
All cases						
Period 1	30.2	<0.001		7.0	<0.001		8.6	0.45
Period 2	25.7			4.4			9.3	
Gender								
Male								
Period 1	43.5	0.01		9.4	0.08		11.9	0.91
Period 2	34.8			6.6			12.0	
Female								
Period 1	18.4	0.70		4.8	0.02		5.6	0.26
Period 2	17.4			2.4			6.9	
Age, y								
<35								
Period 1	28.4	0.25		8.4	0.03		5.4	0.007
Period 2	24.9			5.2			9.4	
35–64								
Period 1	30.2	0.11		5.6	0.41		10.5	0.28
Period 2	24.8			4.3			8.2	
>64 y								
Period 1	32.4	0.52		4.6	0.16		12.5	0.81
Period 2	28.9			2.8			11.4	
Nationality								
Spanish-born								
Period 1	29.9	<0.001		7.2	<0.001		8.6	0.04
Period 2	20.0			3.9			6.3	
Foreign-born								
Period 1	40.0	0.12		1.8	0.23		9.1	0.02
Period 2	58.7			7.3			26.6	

*Period 1, 1997–1999; period 2, 2002–2004. All rates (overall, clustered, and nonclustered) refer to average incidence rates/100,000 persons during per the 3-year study period. †Overall rates refer to all tuberculosis cases. Clustered and nonclustered case rates were calculated only in cases with restriction fragment length polymorphism analysis.

## Conclusions

Our data show a significant decrease in the incidence of clustered TB cases from 1997–1999 to 2002–2004. Clustering in urban areas can be used as a surrogate measure for recent transmission (*3*,*4*). This reduction has had an important epidemiologic effect on the overall TB case rate in Madrid. Among Spanish-born persons, the incidence of clustered and nonclustered cases declined significantly, reflecting a significant decrease in case rate. However, among foreign-born persons, overall TB incidence increased between the 2 study periods; rate of nonclustered cases increased significantly, as did rate of clustered cases, although less dramatically. Both changes are affecting overall incidence of TB in this population.

The relative rates of recent transmission and reactivation of disease have major implications for TB control. Studies in other cities with similar overall case rates have demonstrated that decreasing the incidence of TB is possible if recent transmission is adequately controlled. After implementation of measures to control recent transmission in San Francisco, TB case rate declined from 46.0/100,000 in 1991 to 29.8/100,000 in 1997 (*5*). Similarly, in New York the number of TB cases declined by 65% from 1992 to 2000 after strict measures to identify and treat active cases were introduced (*6*). In contrast, other studies have demonstrated declining rates of disease caused predominantly by reactivation of past infections (*3*,*7*).

Another study in San Francisco (*4*) showed that the intensification of control measures decreased the overall incidence of TB by reducing number of clustered cases, which plateaued at 3 clustered cases/100,000. In our study, the rate of clustered TB cases decreased significantly between the 2 study periods, from 7.0 to 4.4 cases/100,000. We believe that directing control measures toward specific demographic subgroups remains an opportunity for reducing the rate of TB transmission in Madrid.

Overall rate of nonclustered cases did not change over the length of the study; in fact, this rate increased among foreign-born persons, most of whom were <35 years of age. Although we may be underestimating the percentage of foreign-born persons involved in recent transmission (because of the difficulty in surveying this highly mobile population), most cases in persons from countries of origin with high TB endemicity are likely to have been caused by reactivation of TB. As a consequence, greater effort is required to identify and treat latent TB infection in immigrant communities.

In conclusion, incidence of TB in Madrid decreased from 1997 through 2004, predominantly as a result of declining rates of recent transmission among the native Spanish-born population. The reduced incidence of clustered cases coincided with an increased number of TB cases among foreigners and likely indicates introduction of new strains of *M. tuberculosis* into the community. To further control TB, recent transmission of TB must be reduced by intensifying measures to identify contacts. In addition, a strategy is needed for screening for TB infection and case finding among foreign-born persons when they first contact the health system in Spain. Findings such as these from similar long-term, population-based molecular epidemiologic studies can be a powerful tool for continued improvement of TB control programs.
